# Prenatal Alcohol Exposure and Miscarriage, Stillbirth, Preterm Delivery, and Sudden Infant Death Syndrome

**Published:** 2011

**Authors:** Beth A. Bailey, Robert J. Sokol

**Keywords:** Prenatal alcohol exposure, pregnancy, pregnancy outcomes, childbirth, miscarriage, stillbirth, preterm delivery, sudden infant death syndrome, animal studies, human studies, literature review

## Abstract

In addition to fetal alcohol syndrome and fetal alcohol spectrum disorders, prenatal alcohol exposure is associated with many other adverse pregnancy and birth outcomes. Research suggests that alcohol use during pregnancy may increase the risk of miscarriage, stillbirth, preterm delivery, and sudden infant death syndrome. This research has some inherent difficulties, such as the collection of accurate information about alcohol consumption during pregnancy and controlling for comorbid exposures and conditions. Consequently, attributing poor birth outcomes to prenatal alcohol exposure is a complicated and ongoing task, requiring continued attention to validated methodology and to identifying specific biological mechanisms.

As has been detailed throughout this issue, prenatal alcohol exposure is a risk factor for many adverse physical and behavioral outcomes. Growth retardation, including intrauterine growth restriction, facial dysmorphology, and neurodevelopmental problems, are hallmarks of fetal alcohol syndrome (FAS) and also are seen throughout the continuum of fetal alcohol spectrum disorders (FASD). In addition to these well-studied effects, other pregnancy and birth outcomes have been linked to prenatal alcohol exposure. Although not typically regarded as definitive indicators of either FAS or FASD, these complications nonetheless have been shown in both animal and human studies to be associated with prenatal alcohol exposure. This article reviews research examining the role of prenatal alcohol exposure in spontaneous abortion, stillbirth, preterm delivery, and sudden infant death syndrome (SIDS). It provides a broad survey of the relevant literature and presents the most reliable information regarding adverse outcomes associated with prenatal alcohol exposure. The article therefore focuses on findings with sufficient confirmatory data and general consensus regarding the effects of prenatal alcohol exposure and presents only minimal detail on how prenatal alcohol exposure may affect birth outcomes, as there are many theories about potential mechanisms, which vary by outcome, and have not been widely confirmed.

## Research Methods and Limitations

When reviewing studies of the associations between prenatal alcohol exposure and birth outcomes, it is important to understand the limitations inherent in this type of research. First, alcohol consumption data typically are collected via self-report because no reliable biomarker currently is available. Given the increasing awareness of the dangers of drinking during pregnancy and the consequent social stigma, many women who consume alcohol are likely to either deny use or significantly underreport the amount that they drink (Ernhart et al. 1988). Thus, the entire distribution of alcohol consumption may be shifted significantly lower than the actual values (Sokol 1980), making determination of specific consumption thresholds difficult. Consequently, statements about “safe” versus “risk” levels of pregnancy alcohol consumption should be made with caution, and no amount of alcohol consumption should be considered safe. Related to this, an additional concern is that false denial of drinking will result in some women who consume alcohol during pregnancy being classified as nondrinkers. This misclassification can increase type II error (i.e., false negatives) by increasing risk outcomes in the group considered to have no prenatal alcohol exposure, making it less likely that effects that truly are present will be identified. Therefore, it is critical that every effort be made to collect alcohol consumption information using methods that maximize accurate disclosure. Such methods, which include detailed recall of exact amounts over a specified period of time as well as nonjudgmental questioning, have been refined over decades of research and are used in reliable studies of this type.

A second factor affecting research on prenatal alcohol exposure is the recent discovery that some women appear to have an increased susceptibility to the deleterious effects of alcohol use during pregnancy. Thus, what might represent a somewhat “safe” level of pregnancy alcohol consumption for one woman may be incredibly risky for another. Women over the age of 30 appear to have an especially increased risk for poor pregnancy outcomes related to drinking during pregnancy (Sokol et al. 2007), as do women of certain racial and ethnic backgrounds. For example, African American infants have been found to be especially susceptible to adverse effects of prenatal alcohol exposure (Sokol et al. 1986). Research also is beginning to explore genetic markers or mutations associated with increased susceptibility to alcohol’s effects (Warren and Li 2005). Consequently, determining a risk threshold that applies generally is further complicated.

Still another issue to be aware of in studies of prenatal alcohol exposure is how alcohol consumption is quantified. The vast majority of studies record alcohol consumption in terms of the number of drinks. The problem, however, is that different types of alcoholic beverages contain different amounts of alcohol. In addition, even within a type of beverage category, people have different ideas of how many ounces constitute a drink. Research methods have been developed to calculate, as accurately as possible, how much a person is drinking in terms of ounces of absolute alcohol. In this case, a drink is defined as 0.5 ounces (or just over 14 grams) of absolute alcohol. Research questionnaires and interviews ask for specific details about what is consumed, including brand name and the sizes of the containers used for poured drinks. In doing so, the number of drinks consumed is standardized across similar research studies, and most of the current studies that are regarded as reliable and valid use this type of methodology.

Finally, alcohol consumption during pregnancy does not typically occur in a vacuum. Women who drink during pregnancy are significantly more likely to engage in other negative health behaviors, including smoking and illicit drug use (Aliyu et al. 2009; Ebrahim and Gfroener 2003). In addition, pregnant women who drink alcohol are more likely to have had multiple previous pregnancies; to be older, unmarried, and of lower socioeconomic status; to experience greater levels of stress; and to have a comorbid mental health condition (Caetano et al. 1998; Ebrahim et al. 1998; King-Hele et al. 2009; Perreira and Cortes 2006; Rubio et al. 2008). All of these associated factors have been shown to increase the risk of poor pregnancy outcomes, including spontaneous abortion, stillbirth, preterm delivery, and, in some cases, SIDS. Because of this, it becomes difficult to point with certainty to alcohol consumption as a proximate cause of these outcomes, even in studies with rigorous multivariate control, as the increased risk may in fact be a result of less precisely measured comorbid factors rather than a primary causal link (Abel 1997).

Despite the limitations in studies of human prenatal alcohol exposure, findings have been confirmed in animal studies without such limitations, pointing to a causal association between prenatal alcohol exposure and poor pregnancy outcomes and suggesting specific biological mechanisms. However, such control clearly is not possible in human studies. At best, studies can assess alcohol consumption as reliably as possible and attempt to account for confounding factors through specific sample selection or statistical methods of control. This is not always easy, as many of the associated risk factors occur at very high rates in pregnant drinkers. For example, 11.4 percent of pregnant women in the United States smoke cigarettes (Martin et al. 2003), a known risk factor for poor pregnancy outcomes. However, in a recent large-scale epidemiological study, more than 55 percent of women who admitted to drinking during pregnancy were smokers (Aliyu et al. 2009). Thus, it is difficult to sample enough non-smokers who consume alcohol who are truly representative of drinkers in general. Statistical control also presents a problem because after removing variance in pregnancy outcomes attributed to smoking, often very little unique variance remains that can be attributed to alcohol exposure. In addition, as also may be the case for other risk factors, prenatal tobacco exposure has been shown to synergize with prenatal alcohol exposure, together increasing the risk of poor outcomes beyond the sum of the effects of either individually (Odendaal et al. 2009).

Consequently, attributing poor birth outcomes to pregnancy alcohol consumption is a complicated and ongoing task that requires continued attention to validated methodology and to identifying specific biological mechanisms. The studies reviewed in the sections below generally are regarded as being adequately designed to produce valid findings and make at least tentative causal inferences about the association between prenatal alcohol exposure and birth outcomes.

## Spontaneous Abortion

Spontaneous abortion, typically defined as fetal demise prior to 20 completed weeks of gestation, is the most common negative outcome of pregnancy. Although many spontaneous abortions occur before the pregnancy is known, clinically recognizable losses occur in as many as 20 percent of all pregnancies (Kline et al. 1989). The reason for the fetal loss often is unknown; however, in some cases certain risk factors are present. Alcohol exposure during pregnancy has been examined as one potential risk factor for spontaneous abortion. As early as 1980, research evidence suggested that drinking during pregnancy—at least heavy drinking—was associated with a significantly increased rate of spontaneous abortion (Sokol 1980). Women who consumed at least one alcoholic beverage per day during pregnancy had more spontaneous abortions, mainly during the second trimester, than did women who did not drink or drank lesser amounts. Women who consumed more than three drinks daily had a more than threefold increase in risk (Harlap and Shiono 1980).

Recent attention has focused on more moderate levels of alcohol consumption during pregnancy. In a large-scale prospective cohort study (Windham et al. 1997) conducted in California in the early 1990s, women who consumed more than three drinks per week during the first trimester had a significantly increased risk of spontaneous abortion. In another large-scale prospective study (Kesmodel et al. 2002*a*), this one conducted in Denmark, women who consumed five or more drinks per week in the first trimester had a fivefold increase in risk of first-trimester spontaneous abortion. Researchers did not find an association between alcohol intake during the second trimester and spontaneous abortion, however. In another smaller Danish study (Rasch 2003), consumption of five or more drinks per week was associated with a fivefold increase in risk for spontaneous abortion. No association was found between consumption of one to four drinks per week and spontaneous abortion. In all three of these studies, effects remained significant after control for potentially confounding factors, including age, parity, socioeconomic status, and use of other substances.

It appears, then, that alcohol consumption during pregnancy, at least at moderate to heavy levels, is linked to spontaneous abortion. However, a review (Henderson et al. 2007) examining the impact of light to moderate prenatal alcohol exposure concluded that there is no consistent evidence for an increased risk of spontaneous abortion at these lower levels of exposure. The studies described above also seem to support that conclusion. These studies and others would appear to suggest that the risk for spontaneous abortion is not increased unless at least three drinks are consumed per week. However, as we detailed above, caution should be exercised in drawing conclusions about potential alcohol consumption thresholds based on these findings.

## Stillbirth

Fetal demise occurring after 20 weeks gestation, or stillbirth, affects 6.22 of every 1,000 pregnancies in the United States each year (MacDorman et al. 2009). This pregnancy outcome may occur more frequently among those who consume alcohol during pregnancy. Early studies showed that alcohol intake of 14 or more drinks per week during pregnancy was associated with stillbirth (Marbury et al. 1983). Kesmodel and colleagues (2002*b*) demonstrated that consuming more than five drinks per week led to a threefold increase in stillbirth risk, even after adjustment for potentially confounding socioeconomic and lifestyle factors. Animal studies also have demonstrated a fourfold increase in stillbirth rates in conjunction with gestational alcohol administration (Ba 2009).

A study (Aliyu et al. 2008) of more than 600,000 human births found a statistically significant 40 percent increase in likelihood of stillbirth for women who consumed any amount of alcohol compared with those who did not consume alcohol at all. Data were further analyzed to examine the amount of alcohol consumption, and the increased risk was almost completely attributed to those who consumed five or more drinks per week. Interestingly, women who reported drinking during pregnancy but failed to provide information on the amount of consumption had the highest risk of experiencing stillbirth. These women may in fact have represented those with the highest levels of alcohol consumption, providing further support for the hypothesis that responding according to social desirability may have an impact on the accuracy of the data on the amount of alcohol consumption during pregnancy. This study also examined timing of stillbirth. Women were categorized by early stillbirth (prior to 28 weeks’ gestation) or later stillbirth. Pregnancy alcohol consumption was significantly associated with early (any drinking led to an 80 percent increase in risk) but not late (only a nonsignificant 20 percent increase in risk) stillbirth. However, these findings related to stillbirth timing may be a product of the particular study sample, as timing of stillbirth was not an a priori hypothesis, and further research may or may not explicate this particular finding. Regardless, the findings from Aliyu and colleagues (2008) provide additional confirmation of the link between prenatal alcohol exposure and stillbirth described in earlier reports.

Although increased risk for stillbirth among women who consume alcohol may be a result of associated socioeconomic and lifestyle factors, the studies reported above controlled for many of these factors and still found significant effects. Animal studies that have produced associations between prenatal alcohol exposure and stillbirth further support the idea that alcohol exposure is exerting a direct biological effect that results in fetal demise, rather than identified effects being attributed solely to associations between alcohol consumption and other risk factors. Finally, a review (Burd et al. 2007) reported that prenatal alcohol exposure has been shown to be associated with placental dysfunction, decreased placental size, impaired blood flow and nutrient transport, and endocrine changes, any of which could play a role in stillbirth and in the alcohol exposure effects on preterm birth described below.

## Preterm Birth

Preterm birth is typically defined as delivery occurring before 37 completed weeks of gestation. In the United States, there are nearly half a million preterm deliveries each year, or 12.1 percent of all births (Martin et al. 2003). Babies born prior to 37 weeks can be further classified as mildly preterm (32 to 36 weeks) and extremely preterm (less than 32 weeks). Nearly two dozen published studies have examined the potential association between alcohol consumption during pregnancy and preterm birth, and the assertion that a link does indeed exist is controversial (Bailey and Sokol 2008). Many studies suffered from methodological weaknesses, including small sample size, failure to adequately control for confounding factors, inadequate assessment of alcohol exposure, and unreliable gestational dating (i.e., using menstrual cycle dates rather than ultrasound dating), and consequently most reported no significant link between prenatal alcohol exposure and preterm birth (Bailey and Sokol 2008). Two studies (Kesmodel et al. 2000; O’Leary et al. 2009) that primarily relied on ultrasound dating to establish prematurity and controlled for many potentially confounding factors both demonstrated an increased risk of preterm birth associated with prenatal alcohol exposure. In one study (Kesmodel et al. 2000), consumption of 10 or more drinks per week was associated with a nearly threefold increase in the risk of delivery prior to 37 weeks. Consumption at lower rates was not significantly associated with preterm delivery. In the other study (O’Leary et al. 2009), which also found no link between low to moderate alcohol consumption and preterm delivery, binge drinking at any point during pregnancy and heavy drinking during the first trimester both predicted a two-to threefold increase in risk in prematurity. However, some effects fell short of statistical significance after controlling for confounding because of small group sizes.

Two other studies with large samples, adequate control for confounding factors, and reliable assessment of alcohol exposure and gestational dating have examined the link between prenatal alcohol exposure and different levels of prematurity. Adams and colleagues (1995) examined preterm delivery risk factors among a cohort of Army service women. Although gestational age at birth was not universally obtained through ultrasound examination, it was the method available for most women. Alcohol consumption at entry into prenatal care was associated with a significant increase in extreme preterm delivery (29 to 32 weeks’ gestation). Level of alcohol consumption was not considered.

Sokol and colleagues (2007) conducted a large-scale study following over 3,000 women prospectively for pregnancy substance use, with ultrasound-confirmed pregnancy dating. Prenatal alcohol exposure was associated with significantly increased risk of extreme preterm delivery (less than 32 weeks) after controlling for potential confounders, including the use of other substances, demographics, and clinical factors. Specifically, based on risk estimates, if all women in the sample had abstained from alcohol consumption during pregnancy, more than two of every five preterm births would have been avoided. Prenatal alcohol exposure also was associated with mild prematurity but only for women over 30 years of age. The actual amount of alcohol consumption needed in order to see an effect on preterm birth is not clear from this study, as the number of days in which alcohol was consumed each week was analyzed rather than total number of drinks. However, a repeat analysis including women with methods of gestational age dating other than ultrasound failed to detect an association between prematurity and alcohol consumption. Thus, it appears that accurate dating of gestational age examined at different levels of prematurity is crucial in identifying associations between alcohol exposure and preterm birth, and may explain why this effect has not been observed in other studies (Bailey and Sokol 2008). It also appears that, at present, evidence for increased risk in overall preterm birth attributed to low to moderate levels of alcohol consumption during pregnancy is inconsistent (Henderson et al. 2007). This could be caused by many factors, including the fact that being born preterm reduces and/or eliminates alcohol exposure during the third trimester, thus potentially reducing the impact of exposure compared with what might have occurred had the pregnancy gone to term. Additionally, a more definite risk profile does emerge when alcohol consumption at higher levels is examined against different levels of prematurity.

## SIDS

Despite increased awareness and risk-reduction messages, SIDS remains the leading cause of infant mortality in the United States, occurring at a rate of 0.53 per 1,000 infants (Mathews and MacDorman 2006). In 2002, Iyasu and colleagues (2002) published the first report detailing an association between prenatal alcohol exposure and SIDS. Although prenatal tobacco exposure had long been thought to contribute to SIDS, prenatal alcohol exposure had never been independently associated with this outcome. Early pregnancy alcohol consumption at any level was associated with a significantly increased risk of SIDS even after controlling for other potential confounders. Similarly, first-trimester binge drinking also was highly associated with SIDS in the case–control study of 99 Plains Indians infants (Iyasu et al. 2002). Alcohol consumption later in pregnancy was not significantly associated with the incidence of SIDS. In another study, infants who died from SIDS were nearly twice as likely as those who died from other causes to have had any prenatal alcohol exposure (Duncan et al. 2008). This difference was not statistically significant because of the small sample size and number of confounding factors. However, infants in SIDS cases were more than three times as likely to have had exposure to binge drinking prenatally, a difference that did reach statistical significance.

Clearly, more studies with significantly larger samples are needed before definitive conclusions about the association between prenatal alcohol exposure and SIDS can be made, and the Prenatal Alcohol and SIDS and Stillbirth Network is working to develop additional research on this potentially causal link. In addition, recent studies on infant sleep regulatory mechanisms lend further support to the possibility of an association. In one small-scale study (Troese et al. 2008), infants of mothers with the highest alcohol use estimates (median split) exhibited atypical infant sleep state and movement parameters. These infants had increased sleep fragmentation, with brief sleep episodes that were interrupted after sleep onset by wakefulness, and significant reductions in the duration of sleep-related spontaneous movements. Consistent with the resultant developing sleep deprivation, prenatal alcohol exposure also was significantly related to maternal reports of decreased infant alertness and increased irritability. All of these sleep-related parameters have been found to predict increased risk for SIDS in other studies (Schectman et al. 1992).

## Conclusions

Clearly, alcohol consumption during pregnancy has wide-reaching effects. In addition to the more typically seen FAS and FASD outcomes, many other adverse pregnancy and birth outcomes have been linked to prenatal alcohol exposure. Although further research is needed, existing studies suggest that drinking during pregnancy may increase the risk of miscarriage, stillbirth, preterm delivery, and SIDS. It remains to be seen whether these effects primarily are attributed to true biological effects, sociodemographic and lifestyle factors that co-occur with pregnancy drinking, or, most likely, a combination and possibly synergistic effect. Animal studies, tightly controlled human studies, and studies that have examined structural and chemical alterations would suggest, at least in part, a direct physiological mechanism. Thus, it is incumbent upon prenatal care providers to identify and address pregnancy alcohol use with their patients. Brief, reliable screening tools are available, and interventions with pregnant women to reduce or eliminate their alcohol consumption are easy to implement and are known to be effective. With these efforts, health care providers can help to reduce the incidence and consequences of the preventable adverse effects that are attributable to drinking during pregnancy.

## References

[b1-arh-34-1-86] Abel EL (1997). Maternal alcohol consumption and spontaneous abortion. Alcohol and Alcoholism.

[b2-arh-34-1-86] Adams MM, Sarno AP, Harlass FE (1995). Risk factors for preterm delivery in a healthy cohort. Epidemiology.

[b3-arh-34-1-86] Aliyu MH, Wilson RE, Zoorob R (2008). Alcohol consumption during pregnancy and the risk of early stillbirth among singletons. Alcohol.

[b4-arh-34-1-86] Aliyu MH, Wilson RE, Zoorob R (2009). Prenatal alcohol consumption and fetal growth restriction: Potentiation effect by concomitant smoking. Nicotine & Tobacco Research.

[b5-arh-34-1-86] Bâ A (2009). Alcohol and B1 vitamin deficiency-related stillbirths. Journal of Maternal–Fetal & Neonatal Medicine.

[b6-arh-34-1-86] Bailey BA, Sokol RJ (2008). Is prematurity a part of fetal alcohol spectrum disorder?. Expert Review of Obstetrics & Gynecology.

[b7-arh-34-1-86] Burd L, Roberts D, Olson M, Odendaal H (2007). Ethanol and the placenta: A review. Journal of Maternal–Fetal and Neonatal Medicine.

[b8-arh-34-1-86] Caetano R, Ramisetty-Mikler S, Floyd LR (2006). The epidemiology of drinking among women of child-bearing age. Alcoholism: Clinical and Experimental Research.

[b9-arh-34-1-86] Duncan JR, Randall LL, Belliveau RA (2008). The effect of maternal smoking and drinking during pregnancy upon ^3^H-nicotine receptor brainstem binding in infants dying of the sudden infant death syndrome: Initial observations in a high risk population. Brain Pathology.

[b10-arh-34-1-86] Ebrahim SH, Gfroerer J (2003). Pregnancy-related substance use in the United States during 1996–1998. Obstetrics and Gynecology.

[b11-arh-34-1-86] Ebrahim SH, Luman ET, Floyd RL (1998). Alcohol consumption by pregnant women in the United States during 1988–1995. Obstetrics and Gynecology.

[b12-arh-34-1-86] Ernhart CB, Morrow-Tlucak M, Sokol RJ, Martier S (1988). Underreporting of alcohol use in pregnancy. Alcoholism: Clinical and Experimental Research.

[b13-arh-34-1-86] Guntheroth WG, Spiers PS (2002). The triple risk hypotheses in sudden infant death syndrome. Pediatrics.

[b14-arh-34-1-86] Harlap S, Shiono PH (1980). Alcohol, smoking, and incidence of spontaneous abortions in the first and second trimester. Lancet.

[b15-arh-34-1-86] Henderson J, Gray R, Brocklehurst P (2007). Systematic review of effects of low-moderate prenatal alcohol exposure on pregnancy outcome. BJOG.

[b16-arh-34-1-86] Iyasu S, Randall LL, Welty TK (2002). Risk factors for sudden infant death syndrome among Northern Plains Indians. JAMA: Journal of the American Medical Association.

[b17-arh-34-1-86] Kesmodel U, Olsen SF, Secher NJ (2000). Does alcohol increase the risk of preterm delivery?. Epidemiology.

[b18-arh-34-1-86] Kesmodel U, Wisborg K, Olsen SF (2002a). Moderate alcohol intake in pregnancy and the risk of spontaneous abortion. Alcohol and Alcoholism.

[b19-arh-34-1-86] Kesmodel U, Wisborg K, Olsen SF (2002b). Moderate alcohol intake during pregnancy and the risk of stillbirth and death in the first year of life. American Journal of Epidemiology.

[b20-arh-34-1-86] King-Hele S, Webb RT, Mortensen PB (2009). Risk of stillbirth and neonatal death linked with maternal mental illness: A national cohort study. Archives of Disease in Childhood, Fetal and Neonatal Edition.

[b21-arh-34-1-86] Kline J, Stein Z, Susser M (1989). Conception to Birth: Epidemiology of Prenatal Development.

[b22-arh-34-1-86] MacDorman MF, Kirmeyer S (2009). Fetal and perinatal mortality, United States, 2005. National Vital Statistics Reports.

[b23-arh-34-1-86] Marbury MC, Linn S, Monson R (1983). The association of alcohol consumption with outcome of pregnancy. American Journal of Public Health.

[b24-arh-34-1-86] Martin JA, Hamilton BE, Sutton PD (2003). Births: Final data for 2002. National Vital Statistics Reports.

[b25-arh-34-1-86] Mathews TJ, MacDorman MF (2006). Infant mortality statistics from the 2003 period linked birth/infant death data set. National Vital Statistics Reports.

[b26-arh-34-1-86] Odendaal HJ, Steyn DW, Elliott A, Burd L (2009). Combined effects of cigarette smoking and alcohol consumption on perinatal outcome. Gynecologic and Obstetric Investigation.

[b27-arh-34-1-86] O’Leary CM, Nassar N, Kurinczuk JJ, Bower C (2009). The effect of maternal alcohol consumption on fetal growth and preterm birth. BJOG.

[b28-arh-34-1-86] Perreira KK, Cortes KE (2006). Race/ethnicity and nativity differences in alcohol and tobacco use during pregnancy. American Journal of Public Health.

[b29-arh-34-1-86] Rasch V (2003). Cigarette, alcohol, and caffeine consumption: Risk factors for spontaneous abortion. Acta Obstetricia et Gynecologica Scandinavica.

[b30-arh-34-1-86] Rubio DM, Kraemer KL, Farrell MH, Day NL (2008). Factors associated with alcohol use, depression, and their co-occurrence during pregnancy. Alcoholism: Clinical and Experimental Research.

[b31-arh-34-1-86] Schectman VL, Harper RM, Wilson AJ, Southall DP (1992). Sleep state organization in normal infants and victims of sudden infant death syndrome. Pediatrics.

[b32-arh-34-1-86] Sokol RJ (1980). Alcohol and spontaneous abortion. Lancet.

[b33-arh-34-1-86] Sokol RJ, Ager J, Martier S (1986). Significant determinants of susceptibility to alcohol teratogenicity. Annals of the New York Academy of Sciences.

[b34-arh-34-1-86] Sokol RJ, Janisse JJ, Louis JM (2007). Extreme prematurity: An alcohol-related birth effect. Alcoholism: Clinical and Experimental Research.

[b35-arh-34-1-86] Sokol RJ, Miller SI, Reed G (1980). Alcohol abuse during pregnancy: An epidemiologic study. Alcoholism: Clinical and Experimental Research.

[b36-arh-34-1-86] Troese M, Fukumizu M, Sallinen BJ (2008). Sleep fragmentation and evidence for sleep debt in alcohol-exposed infants. Early Human Development.

[b37-arh-34-1-86] Warren KR, Li TK (2005). Genetic polymorphisms: Impact on the risk of fetal alcohol spectrum disorders. Birth Defects Research. Part A, Clinical and Molecular Teratology.

[b38-arh-34-1-86] Windham GC, Von Behren J, Fenster L (1997). Moderate maternal alcohol consumption and risk of spontaneous abortion. Epidemiology.

